# Mucor Osteomyelitis of the Distal Radius Necessitating Ulnocarpal Fusion

**DOI:** 10.7759/cureus.12813

**Published:** 2021-01-20

**Authors:** Ekaterina Tiourin, Melissa Kanack, Wendy Ng, Amber Leis

**Affiliations:** 1 Plastic Surgery, University of California, Irvine School of Medicine, Orange, USA; 2 Plastic Surgery, Children’s Hospital of Orange County, Orange, USA

**Keywords:** distal radius, fungal hand infection, mucor osteomyelitis, ulnocarpal fusion

## Abstract

This is a case report of a 60-year-old female who developed distal radius osteomyelitis secondary to *Mucor* infection from likely hematogenous spread that was managed with ulnocarpal wrist fusion. Following serial debridement and systemic antifungal therapy, ulnocarpal wrist fusion offered functional limb salvage rather than amputation in this patient with significant operative risk and comorbidities.

## Introduction

Osteomyelitis arising from fungal infection is rare, accounting for less than 10% of the cases [1]. Fungal osteomyelitis is most commonly associated with *Candida* and *Aspergillus* species and rarely with *Mucor* [2]. The most common reported sites of invasive mucormycosis are the sinuses (39%), lungs (24%), and skin (19%), originating from inhalation of spores versus direct inoculation into the disrupted skin or mucosa [3]. Osteomyelitis secondary to mucormycosis has been most commonly associated with trauma or surgical intervention [2,4,5]. Hematogenous spread of mucormycosis causing osteomyelitis is exceedingly rare [5,6]. Reported cases of upper extremity bone infections with mucormycosis have been limited to contiguous extension from diseased cutaneous soft tissue [7,8]. We report the case of a 60-year-old female with advanced cirrhosis and diabetes who developed distal radius osteomyelitis secondary to mucormycosis infection from likely hematogenous spread that was managed with ulnocarpal wrist fusion.

## Case presentation

**Figure 1:**
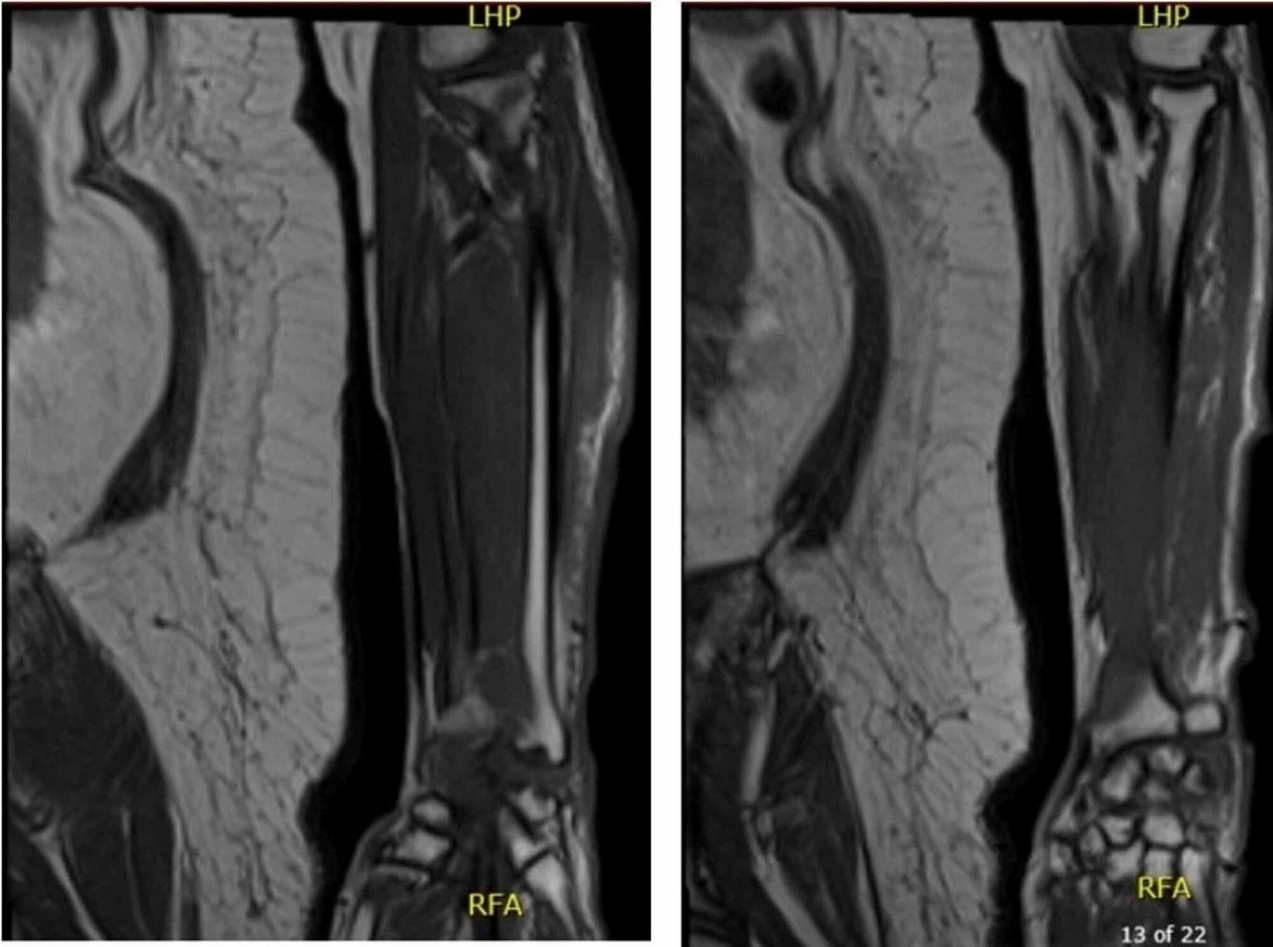
MRI with contrast of the left forearm showing cortical bone destruction and bone marrow edema with enhancing endosteum/periosteum of the radius diaphysis and metaphysis. MRI, magnetic resonance imaging

A 60-year-old, left-hand dominant female with cirrhosis (Model for End-Stage Liver Disease [MELD] score 16) and type II diabetes mellitus on insulin presented to the emergency room with left forearm burning, pain, and swelling. She denied any history of trauma or new exposure to the hand. After receiving a diagnosis of cellulitis and a course of cephalexin, she returned four days later with fever as well as increased dorsal hand swelling and erythema. Computed tomography (CT) imaging demonstrated no evidence of necrotizing soft tissue infection and abscess. She did not present any motor or sensory deficits of the digits or wrist. She was prescribed a course of ceftriaxone and vancomycin. One month later, she presented again to the emergency room for altered mental status. While receiving treatment for hepatic encephalopathy, she underwent imaging of her left forearm due to continued pain. CT imaging now showed diffuse patchy lucencies and severe cortical thinning of the distal radius with a differential diagnosis of osteomyelitis, bone infarct, or aggressive osteopenia. Contrast-enhanced magnetic resonance imaging further showed significant cortical bone destruction as well as bone marrow edema with enhancing endosteum and periosteum consistent with osteomyelitis of the distal radius (Figure 1).

**Figure 2:**
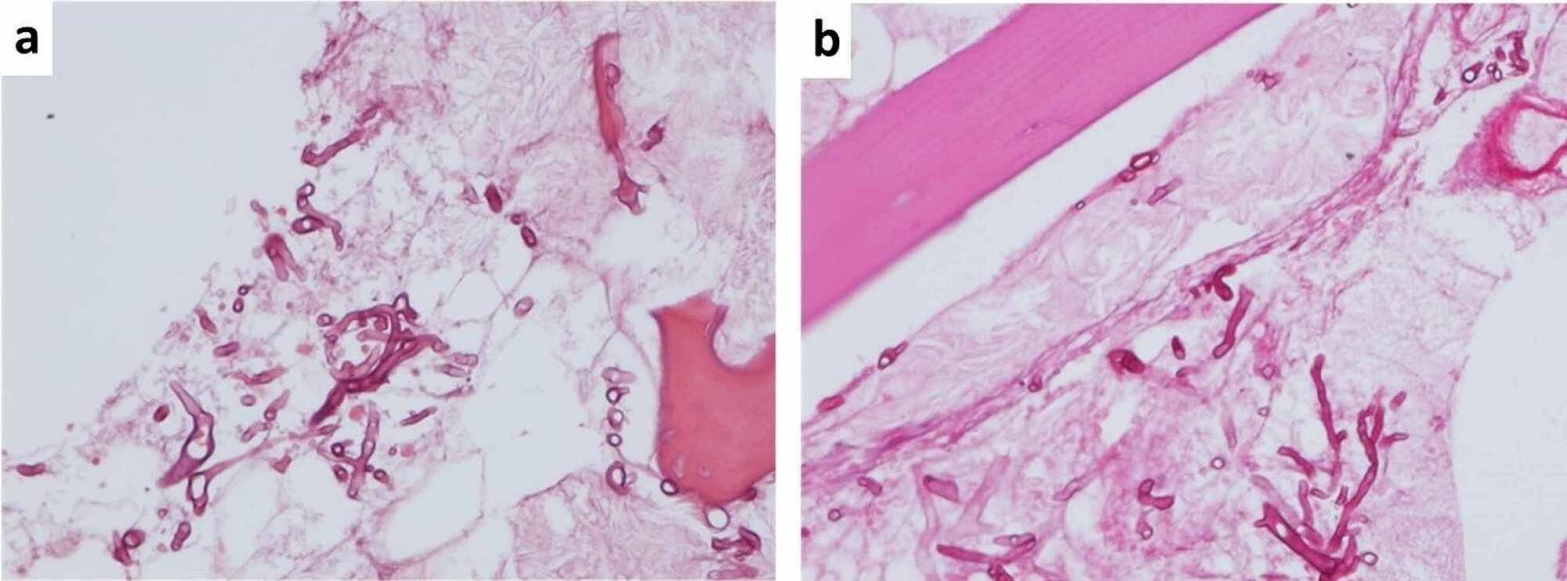
(A) H&E stain of the distal radius biopsy. (b) PAS stain of the distal radius biopsy. H&E, hematoxylin and eosin; PAS, periodic acid-Schiff

There was no overlying soft tissue ulceration or fluid collection, suggesting a potential hematogenous spread of infection. A CT-guided biopsy confirmed bone necrosis inundated with fungal hyphae, which was morphologically consistent with *Mucor* via Grocott methenamine silver and periodic acid-Schiff staining (Figure 2).

The infectious disease and hand surgery teams were consulted for management of *Mucor* osteomyelitis of the left distal radius with no evidence of direct inoculation.

**Figure 3:**
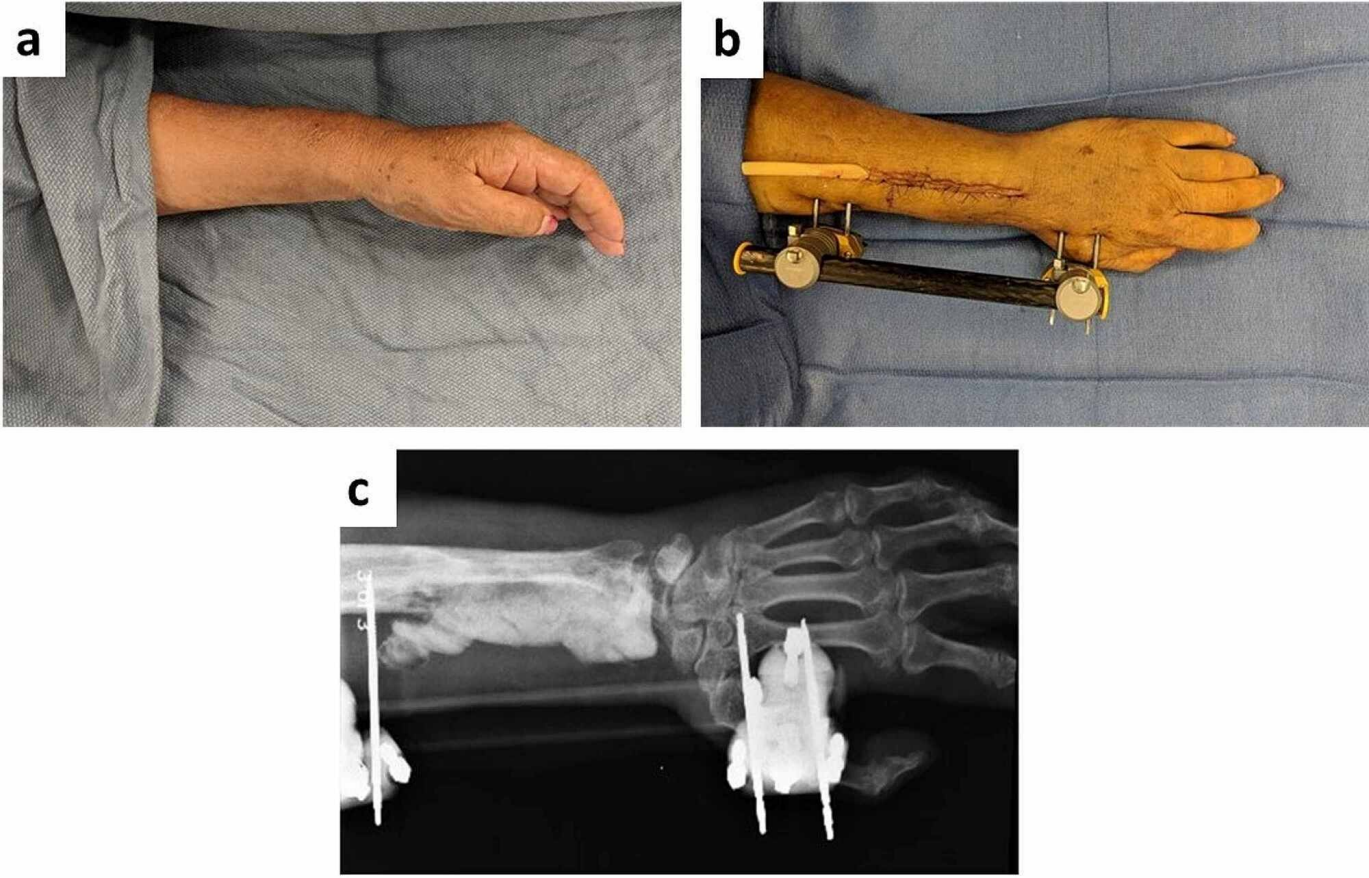
(A) Preoperative image of the left forearm. (B) Postoperative image of the left forearm with external fixator in place after excision of the distal radius, radial shaft, base of lunate, and proximal radius and placement of amphotericin beads. (C) Postoperative X-ray of the left forearm with external fixator in place and placement of amphotericin beads.

She was immediately started on amphotericin. Her first surgical intervention entailed irrigation and debridement of the necrotic radius extending from the radiocarpal joint (including a portion of the lunate) to the proximal radial shaft via a dorsal approach. The bony defect was filled with Stimulan® (Biocomposites, Keele, UK) anti-fungal cement beads. A uni-plane external fixator was placed from the mid-radius to the second metacarpal to span the bony defect while the infection was managed (Figure 3).

She returned to the operating room one week later for repeat irrigation and debridement followed by application of a Stimulan® amphotericin polymethyl methacrylate cement bone spacer contoured to the lunate distally and the diaphysis of the radius proximally. Postoperatively, the range of motion of her left digits were limited though her sensation to light touch remained intact. While recovering, she experienced significant acute kidney injury from amphotericin therapy and was transitioned to isavuconazole treatment. The plan was for more definitive reconstruction involving internal fixation. However, the patient was lost to follow-up as she sought care for her hepatic encephalopathy and extended-spectrum beta-lactamase bacteremia secondary to a urinary infection.

**Figure 4:**
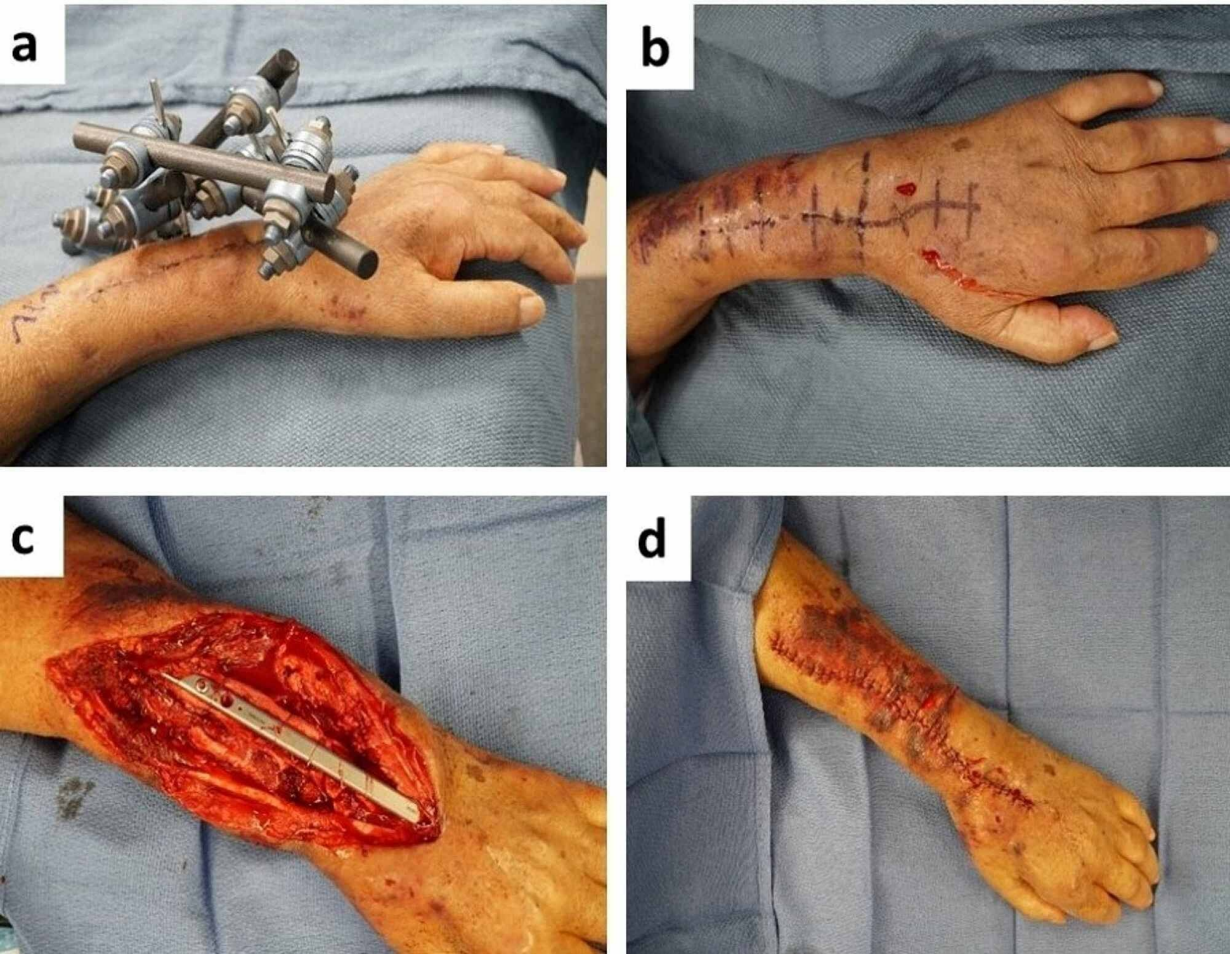
(A) Preoperative image with external fixator in place. (B) Preoperative image with external fixator removed. (C) Intraoperative placement of spanning bridge plate from the ulna to the third metacarpal. (D) Postoperative closure after distal wrist fusion.

Six months after the placement of the antifungal cement bone spacer, she presented again to the emergency room with left elbow pain and forearm tenderness. X-ray imaging showed inadequate alignment of the carpus and distal radius/ulna with extrusion of the antifungal spacer that was no longer communicating with the radius. She underwent repeat irrigation and debridement, removal of the bone cement, and replacement of the uni-plane fixation with a multi-plane external fixator. At this time, her liver condition had decompensated to a MELD score of 22, which correlated with a 30-day mortality greater than 50% with a major operation. Due to her significant comorbidities and chronic immunosuppression, she was not a candidate for an osseous free flap or cadaveric tissue implant to reconstruct the distal radius. A distal ulnocarpal wrist fusion was performed with a spanning bridge plate from the ulna to the third metacarpal and an iliac crest bone graft (Figure 4).

Her long-term postoperative course was complicated by cirrhosis, acute renal failure, and uncontrolled diabetes. Eleven months postoperatively from wrist fusion, her wounds were well healed. Despite occupational therapy and thermoplastic splinting, she developed significant digital stiffness with limited flexion and extension at the metacarpophalangeal and interphalangeal joints. She retained sensation to light touch in the median, ulnar, and radial nerve distributions. She now uses the left forearm as a helper hand to her right counterpart during her daily activities. Per infectious disease recommendations, she was treated with isuvaconazole for 12 months.

## Discussion

Mucormycosis is a rare but aggressive fungal infection that preferentially affects immunocompromised hosts, such as those with diabetes, transplants, cancers, and iron overloaded conditions [5]. With impaired function of macrophages and neutrophils, immunocompromised hosts are unable to fend off *Mucor* infection [4]. The infective form of mucormycosis exists as sporangiospores that can be inhaled or directly inoculated via the skin or mucosa. When the sporangiospores enter the body, they germinate into the hyphae form that invades blood vessels causing infarction and surrounding tissue necrosis. Without treatment, mucormycosis infection can be lethal [3]. Active suspicion and prompt intervention are crucial.

The origin and spread of *Mucor* infection in this patient remain unclear. Though she denied any trauma to the arm, including any recent burns, scratches, or other lesions, she had a history of hepatic encephalopathy and must be regarded as a poor historian. Upon presentation to the emergency room, however, there was also no sign of an impaired cutaneous barrier of her left arm. Imaging further showed no evidence of skin ulceration or abscess. In severely immunocompromised patients, *Mucor* infection may spread hematogenously [3,6]. It is possible that the patient’s initial presentation of forearm tenderness, swelling, and erythema was consistent with cellulitis. The cellulitis may have caused increased pressure on the underlying bone and potential disruption of leukocytes that collectively made the distal radius more susceptible to hematogenous seeding of *Mucor* [2,4]. In the setting of daily antifungal therapy initiated immediately after diagnosis, she did not develop any signs of disseminated infection beyond the left arm and her blood cultures remained negative.

Cutaneous mucormycosis has been reported in the upper extremity, including the hand [7,8]. In these cases, direct inoculation via existing wounds in mostly immunocompromised patients was the source of infection [7,8]. Treatment modalities implemented for cutaneous mucormycosis have included topical and systemic antifungal therapy, surgical debridement, and hyperbaric oxygen therapy [7,8]. In cases of mucormycosis osteomyelitis at other sites, treatment has also involved surgical debridement and systemic antifungal therapy [2,3,6]. There has been no defined treatment of mucormycosis osteomyelitis of the hand or forearm. Our treatment plan consisted of serial cultures, imaging, involvement of infectious disease experts for antifungal therapy, and surgical intervention. Given the significant comorbidities of the patient and the necessity of infection control, our surgical plan proceeded in a stepwise manner with the goal of limb salvage rather than amputation. We performed several surgical debridements to remove necrotic tissue while implementing antifungal structural support at the site of the bony defect as a bridge to more definitive reconstruction in the future. Though our center and surgeons are well experienced to perform allograft bone free flap reconstruction or cadaveric bone grafting, these strategies were curtailed due to the patient’s decompensation with her comorbidities. Nevertheless, we were able to salvage her limb by performing a distal wrist fusion that provided her with a support to her right hand during her activities of daily living.

In this patient with significant operative risk and comorbidities, ulnocarpal fusion was the most reasonable option for limb salvage. Wrist fusion has been reported as an effective surgical intervention for advanced arthritis, congenital absence of radius, trauma, nerve injuries, and giant cell tumors [9,10]. Ulnocarpal fusion is a relatively short operation that produces a painless stable wrist without the potential complications of grafts [10]. Normal function of the elbow and shoulder help compensate for the loss of wrist movements and facilitate the fused wrist to act as a helper hand to the contralateral limb. Limb salvage helps mitigate the psychological impact of amputation that can further reduce a patient’s quality of life. Ulnocarpal wrist fusion may be the most viable surgical intervention to salvage rather than amputation of the hand and forearm in future cases of mucormycosis osteomyelitis in patients with significant comorbidities.

## Conclusions

Surgery plays a critical role in the salvage of the upper extremity infected with invasive mucormycosis. Surgical intervention is only one part of the multidisciplinary approach to mucormycosis management that must involve infectious disease experts, medical management of comorbidities, and physical therapy to help maintain function of the affected hand. With prompt diagnosis and implementation of this multidisciplinary approach, a hand infected with invasive mucormycosis may achieve a more functional outcome than imminent amputation.
